# PD-1 Inhibitors Combined with Chemotherapy versus Re-irradiation/chemoradiotherapy for Unresectable Locally Recurrent T3-4 Nasopharyngeal Carcinoma: A Retrospective Study

**DOI:** 10.7150/jca.98775

**Published:** 2024-08-26

**Authors:** Tong-Xin Liu, Quan-Quan Sun, Yong-Hong Hua, Chang-Juan Tao, Feng Jiang

**Affiliations:** 1 Department of Radiation Oncology, Zhejiang Cancer Hospital, Hangzhou, Zhejiang, China.; 2 Zhejiang Key Laboratory of Radiation Oncology, Zhejiang Cancer Hospital, Hangzhou, Zhejiang, China.

**Keywords:** Recurrent nasopharyngeal carcinoma, PD-1, Re-irradiation

## Abstract

**Objective:** To evaluate the efficacy, toxicity, and long-term outcomes of PD1 inhibitors plus chemotherapy versus re-irradiation/chemoradiotherapy in patients with unresectable locally recurrent T3-4 nasopharyngeal carcinoma (NPC).

**Methods:** A retrospective analysis was conducted on 42 patients with recurrent nasopharyngeal cancer (NPC) after receiving immunochemotherapy or re-irradiation between February 2018 and May 2022 in Zhejiang Cancer Hospital. Overall survival (OS), progression-free survival (PFS), local recurrence-free survival (LRFS), and distant metastasis-free survival (DMFS) were determined using the Kaplan-Meier method, log-rank test, and Cox proportional hazard regression.

**Results:** With a median follow-up duration of 28.7 months (ranging from 7.2 to 63.9 months), the 3-year OS rate was 23.3% in the re-irradiotherapy (RI) group (N = 24) and 59.6% in the immunochemotherapy (IC) group (N = 18) (p = 0.042). The 3-year PFS, LRFS, and DMFS rates were not significantly different between the two groups (PFS: 45.3% vs. 62.6%, P = 0.482; LRFS: 54.4% vs. 62.6%, P =0.891; DMFS: 89.8% vs. 100.0%, P = 0.489). The univariate analysis revealed that regimen (HR: 0.354, 95% CI: 0.130-0.962, P = 0.042) was significantly correlated with OS. Multivariate analysis also showed that treatment regimen (HR: 0.329, 95% CI: 0.12-0.970, P =0.044) was the only significant prognostic factor associated with OS. The most common late toxicities in the RI group were xerostomia, deafness, and nasopharyngeal necrosis. Of these, nasopharyngeal necrosis was present in 16 patients (66.7%) and in 10 patients (41.7%) at a grade 3 or above. Nasopharyngeal necrosis is the main cause of death in the RI group. In contrast, in the IC group, grade 3 or higher immune-related adverse events or late adverse events were not observed.

**Conclusions:** For unresectable locally recurrent NPC, re-irradiation is an effective treatment; nevertheless, the survival obtains are usually surpassed by serious late complications. For these individuals, chemotherapy in addition to an anti-PD-1 checkpoint inhibitor may be a helpful course of treatment.

## Introduction

Nasopharyngeal carcinoma (NPC) is a kind of malignant tumor originating from the nasopharyngeal epithelium that is common in East and Southeast Asia 1. According to the Global Cancer Statistics 2020, there were about 133,354 new cases of NPC, accounting for 0.7% of all cancers diagnosed, and 80,008 new death cases of NPC, accounting for 0.8% of all cancer deaths in 2020 2. Radiotherapy or chemoradiotherapy is the primary modality for NPC, and the 5-year overall survival rate is approximately 85-90% 3-5. However, despite the implementation of intensity-modulated radiotherapy (IMRT), local recurrence still occurs in 10%-20% of NPC patients after previous radical radiotherapy 6,7.

According to the results of a randomized, controlled Phase Ⅲ clinical trial reported by Liu et al. 8, in patients with resectable locally recurrent NPC, endoscopic nasopharyngectomy (ENPG) significantly improved overall survival compared with IMRT. This study suggested that ENPG might be considered as a standard treatment choice for resectable locally recurrent NPC. International Recommendations on Reirradiation by Intensity Modulated Radiation Therapy for Locally Recurrent Nasopharyngeal Carcinoma also proposed that if expertise was available and clear margins were likely to be achievable, surgical resection was preferred for resectable recurrence of NPC 9. Whereas, many locally recurrent NPC patients are presented with advanced disease that is not amenable to surgery. In such cases, re-irradiation remains the most effective method for recurrent NPC patients. Furthermore, for patients with bulky rT2 stage and rT3-4 stage, induction chemotherapy combined with irradiation or concurrent chemoradiotherapy may facilitate better sparing of adjacent organ at risk (OAR) by shrinking the tumor size and prolonging the time for recovery, especially for those patients with a latency of recurrence less than 12 months. However, due to the severe toxicities of re-irradiation, such as mucosal necrosis, temporal lobe necrosis, trismus, and cranial neuropathy 10, a large proportion of NPC patients die of complications 11,12.

As we know, programmed cell death ligand 1 (PD-1) is highly expressed in EBV-related NPCs 13. Recently, several phase Ⅲ clinical trials have confirmed that PD-1 antibody combined with chemotherapy provides more benefits in the first-line treatment of recurrent or metastasis (R/M) NPC than chemotherapy alone, with a median progression-free survival (PFS) of 9.6-21.4 months 14-16. For patients with recurrent or metastatic NPC, immunotherapy with chemotherapy has thus emerged as the preferred course of treatment. These trials, particularly those including patients with unresectable T3-4 recurrent NPC, did not, however, stratify the effectiveness of immunotherapy in conjunction with chemotherapy for recurrent NPC.

Thus, the aim of this study is to analyze the efficacy and safety of re-radiotherapy or chemoradiotherapy versus an anti-PD1 checkpoint inhibitor combined with chemotherapy in patients with unresectable recurrent T3-4 NPC.

## Methods

### Patient selection

This retrospective study analyzed patients with locally recurrent rT3-4N0-2M0 NPC who were unable to undergo surgery. They received re-radiotherapy, chemoradiotherapy, or PD-1 inhibitors combined with chemotherapy at Zhejiang Cancer Hospital from February 2018 to May 2022. The inclusion criteria for this study were as follows: (1) histologically confirmed NPC, (2) age range from 18 to 80, (3) Karnofsky Score ≥70, (4) no evidence of distant metastases, (5) a time interval from completion of primary radiotherapy to the diagnosis of recurrence (TI) >12 months, (6) rT3-4 locally recurrence, which is not amenable to surgery, (7) adequate organ function, (8) free from other malignant diseases. All patients involved in this research were restaged using the TNM staging system, which is the eighth edition of the International Union Against Cancer/American Joint Committee on Cancer. This research was approved by the Research Ethics Committee of Zhejiang Cancer Hospital.

### Chemotherapy and immunotherapy

There is no standard protocol for induction chemotherapy. Induction chemotherapy consists of the following regimens: GP: gemcitabine (1,000 mg/m^2^, d1,8) and cisplatin or nedaplatin (80 mg/m^2^, d1); TP: docetaxel (75 mg/m^2^, d1) or nanoparticle albumin-bound paclitaxel (260 mg/m^2^, d1) and cisplatin or nedaplatin (80 mg/m^2^, d1); PF: 5-fluorouracil (1g/m^2^, d1-4) and cisplatin (80 mg/m^2^, d1). All the regimens were performed every 21 days for a total of 3-6 cycles. In the re-irradiation group, patients received 3-6 cycles of induction chemotherapy followed by reirradiation or concurrent chemoradiotherapy. The concurrent chemotherapy regimen is cisplatin (80 mg/m^2^, d1) in a cycle of 21 days for 1-2 cycles. In the immunochemotherapy group, anti-PD1 checkpoint inhibitors include toripalimab (240 mg/d), camrelizumab (200 mg/d), and tislelizumab (200 mg/d). Patients received 3-6 cycles of induction chemotherapy combined with an anti-PD1 checkpoint inhibitor, followed by an anti-PD1 checkpoint inhibitor alone every 21 days as maintenance therapy until disease progression, unacceptable toxicity, or the patient's refusal.

### Radiotherapy

In the re-irradiation group, either intensity-modulated radiotherapy (IMRT) or tomotherapy (TOMO) was used for radiation. The nasopharyngeal tumor and positive retropharyngeal lymph nodes, which were established by nasopharyngoscopy and magnetic resonance imaging (MRI), were considered the gross tumor volume of the nasopharynx (GTVnx). The clinical tumor volume (CTV) was defined as an area of 5 mm outside the GTV. The margin may be trimmed appropriately if the tumor is close to critical organs at risk (OARs). The planning target volume (PTV) was defined as CTV or GTV with a 3mm margin. The prescribed doses were as follows: 60-70 Gy to the GTVnx PTV; 50-54 Gy to the CTV PTV. There are two kinds of fractions: one is the conventional fraction, and the fractional dose was 1.8-2 Gy/Fr. The other is hyperfraction, and the fraction for the bid schedule is 1.1-1.2 Gy/Fr (6-h interfraction interval). The dose constraints on the critical structures depend on the disease-free interval (DFI) from the end of primary radiotherapy to recurrence, the maximum tolerated dose, and the Karnofsky score of the patients.

### Outcome and follow-up

The primary endpoint of this study was the objective response rate (ORR). The ORR was assessed using Response Evaluation Criteria in Solid Tumors (RECIST) version 1.1 17. The second endpoint included progression-free survival (PFS), overall survival (OS), and toxicity. Patients were evaluated and followed up after completion of the treatment every 3 months during the first 3 years and every 6 months thereafter. Radiation toxicities were measured according to the radiation morbidity scoring criteria of the Radiation Therapy Oncology Group (RTOG) and the Common Terminology Criteria for Adverse Events (CTCAE) v4.0. The follow-up assessments included patient history, physical examination, plasma Epstein-Barr virus (EBV) DNA, nasopharyngoscopy, MRI examination for the nasopharyngeal and neck, chest CT, and ultrasonography of the abdomen or CT. A whole-body bone scan or Positron Emission Tomography-Computed Tomography (PET-CT) was performed if necessary. The follow-up time was defined as the time from the diagnosis of recurrent NPC until the date of death or the date of the last follow-up. The final follow-up time was in December 2023, with a median follow-up period of 28.7 months (ranging from 7.2 to 63.9 months).

### Statistical analysis

Statistical analyses were performed using SPSS v20.0 software (SPSS, Chicago, IL, USA). The chi-square test or Fisher's exact test was used to compare the characteristics of patients, and the Kaplan-Meier method was used to analyze the OS rate and PFS rate, and the Cox proportional hazards model was used to estimate the multivariate analysis. All statistical tests were two-sided, and *P* < 0.05 indicated a statistically significant difference.

## Results

### Patient characteristics

**Figure 1:**
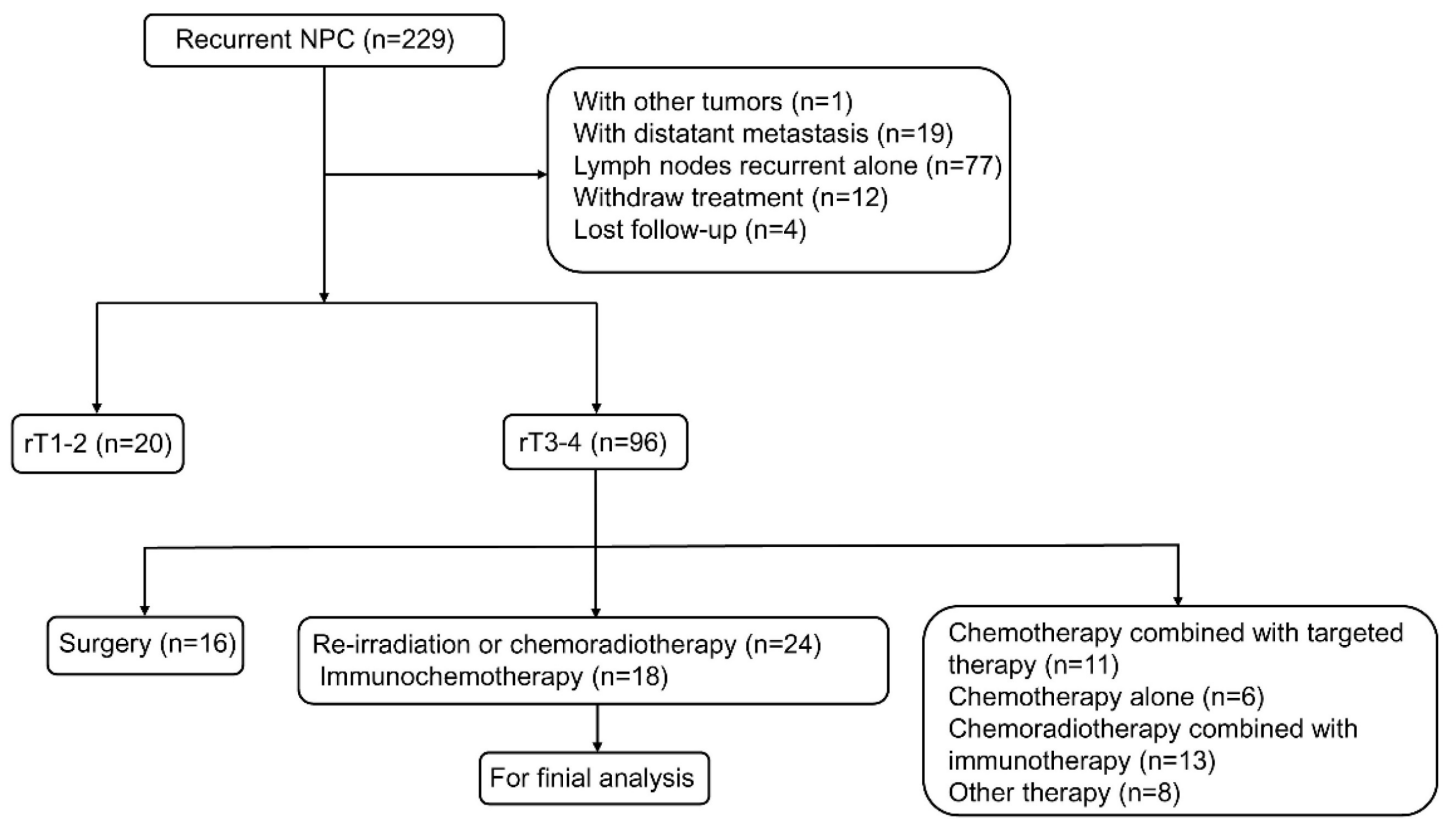
Flow chart showing selection of patients in this study.

From February 2018 to May 2022, a total of 229 patients with locally recurrent NPC were eligible for assessment. Of these, 187 were excluded (Figure 1). Finally, 42 patients were included in the analysis, including 24 patients in the re-irradiation (RI) group and 18 patients in the immunochemotherapy (IC) group. The baseline characteristics of each group were shown in Table 1. The following variables were not statistically different between the two groups: age, gender, KPS score, histology, primary T stage, N stage, and overall stage, TI, recurrence N stage, and EBV DNA copies (all P-values >0.05). However, patients in the IC group had a more advanced recurrence T stage and overall stage compared to those in the RI group (*P*-values < 0.05).

### Efficacy

Tumor response was assessed using RECIST criteria: complete response (CR), partial response (PR), stable disease (SD), and disease progression (PD). In the RI group, 6 patients (25.0%) achieved CR and 16 patients (66.7%) achieved PR; the response rate was 91.7%. While 2 patients (11.1%) achieved CR and 8 patients (44.4%) achieved PR, and the response rate was 55.5% in the IC group (Table 2). The tumor response in the RI group was better than in the IC group.

### Toxicity

The adverse events that occurred in the RI and IC groups are listed in Table 3. In both groups, the most common acute toxic reactions included anemia, leucopenia, neutropenia, thrombocytopenia, and nausea. In addition, the RI group had a higher incidence of dermatitis and stomatitis (mucositis) due to re-irratidaton. The most common acute immune-mediated adverse events were mild to moderate hypothyroidism, pruritus, and fever in the IC group. No Grade 3 or higher immune-associated adverse events occurred.

The most common late toxicities in the RI group were xerostomia, deafness, and nasopharyngeal necrosis. Among them, 16 patients (66.7%) had nasopharyngeal necrosis, and 10 patients (41.7%) had grade 3 or higher nasopharyngeal necrosis. Nasopharyngeal necrosis is the main cause of death in the RI group. In the IC group, the most common late toxicities were mild to moderate xerostomia, deafness, and nasopharyngeal necrosis. Grade 3 or higher late adverse events were not observed.

### Prognosis

**Figure 2:**
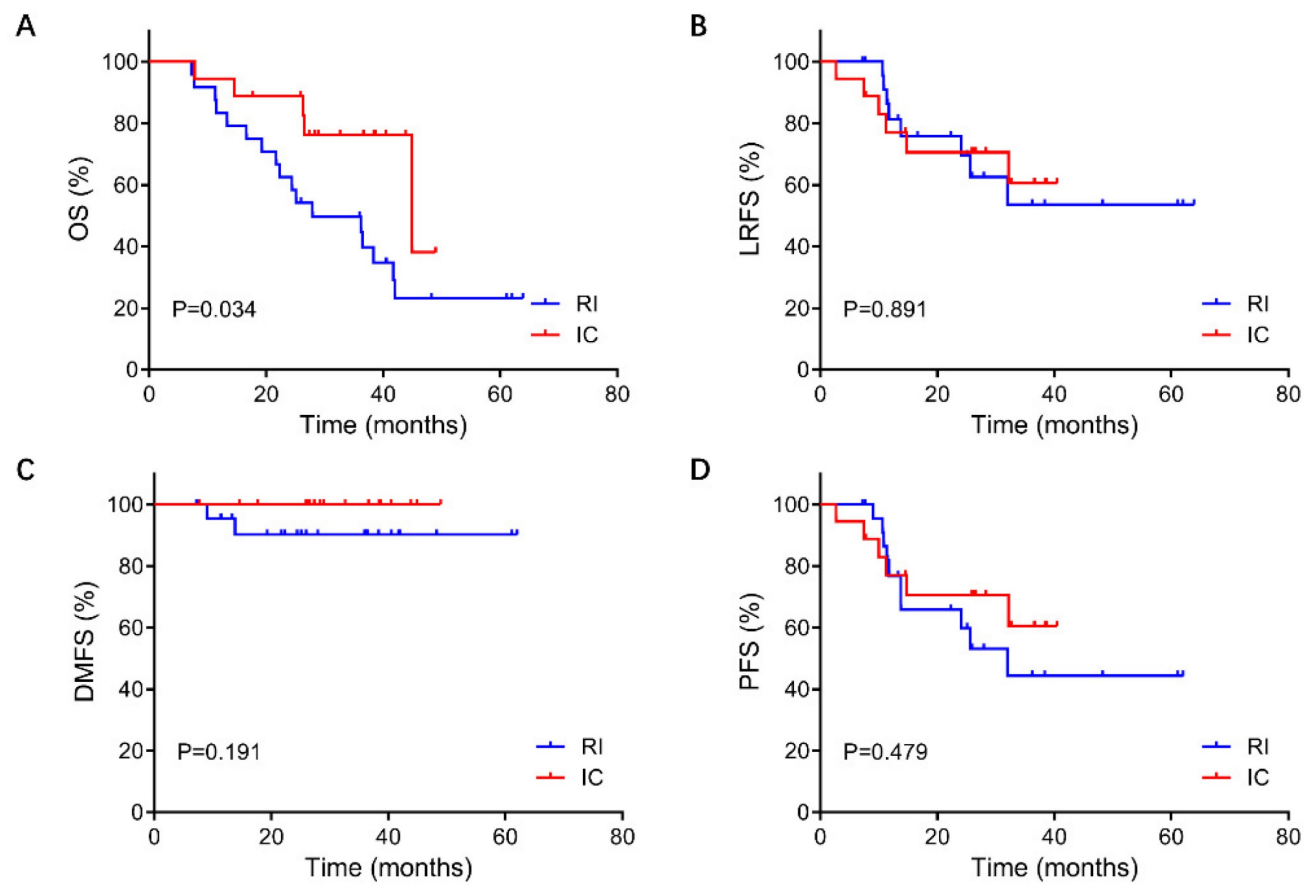
Comparison of survival between re-irradiation and immunochemotherapy group in patients with unresectable recurrent T3-4 nasopharyngeal carcinoma. A: OS, overall survival; B: LRFS, local relapse-free survival; C: DMFS, distant metastasis-free survival; D: PFS, progression-free survival. RT, re-irradiation group; IC, immunochemotherapy group.

For the 42 patients in this study, the median follow-up duration was 28.7 months (ranging from 7.2 to 63.9 months). The 3-year OS rate was 23.3% in the RI group and 59.6% in the IC group (p=0.042). The OS rate of the IC group was significantly better than that of the RI group. In contrast, the 3-year PFS, LRFS, and DMFS rates were not significantly different between the two groups. (PFS: 45.3% vs. 62.6%, P = 0.482; LRFS: 54.4% vs. 62.6%, P = 0.891; DMFS: 89.8% vs. 100.0%, P = 0.489) (Figure 2).

To determine which factor influences patient outcomes, we used univariate analyses to assess the prognostic value of gender, age, histology, primary T stage, primary N stage, primary overall stage, TI, recurrent T stage, recurrent N stage, recurrent overall stage, EBV DNA copies, and regimen. The results revealed that treatment regimen (HR: 0.354, 95% CI: 0.130-0.962, P = 0.042) was significantly correlated with OS (Table 4). Multivariate analysis also showed that treatment regimen (HR: 0.329, 95% CI = 0.112-0.970, P =0.044) was the only significant prognostic factor associated with OS (Table 5).

## Discussion

For early, locally recurrent NPC, endoscopic surgery is the recommended treatment modality 8. However, it can only be used for minor, superficial lesions in specific sites. According to Li et al., only about 25% of NPC patients with initial IMRT developed local recurrence disease at the rT1-2 stage 18. For most NPC patients with unresectable local recurrence, re-irradiation remains the only curative option. A meta-analysis demonstrated that rT1-2 NPC had a 5-year local failure-free survival (LFFS) of 85% (95% CI, 79-91%), a 5-year distant failure-free survival (DFFS) of 95% (95% CI, 90-99%), and a 5-year OS of 62% (95% CI, 51%-74%). In contrast, rT3-4 NPC IMRT had lower rates with a corresponding 5-year LFFS of 68% (95%CI, 61-76%), a 5-year DFFS of 82% (95%CI, 80-84%), and a 5-year OS of 38% (95%CI, 34-42%) 19. Tian et al. also reported that patients with rT3-T4 NPC achieved only a modest local-regional failure-free survival (LRFFS) rate of 60.9% and DFFS of 78.3%, along with an OS rate of 27.5% 10. The limited survival benefit of re-irradiation for patients with unresectable locally recurrent NPC may be attributed to unsatisfactory dose distribution caused by the large size and extensive invasion of the recurrent tumor, as well as constraints on organs at risk. Additionally, the radioresistance of recurrent NPC and the high toxicity associated with re-irradiation could also contribute to this limitation. Severe late complications, such as necrosis of the nasopharyngeal mucosa, hemorrhage, temporal lobe necrosis, and cranial neuropathy, are the primary causes of mortality in patients with recurrent NPC after re-irradiation. These patients can achieve a reduction in gross tumor volume by induction chemotherapy, thereby reducing the clinical tumor volume and facilitating target coverage and dose distribution while decreasing the dose delivered to adjacent critical structures. In addition, induction chemotherapy can also be used as a bridge to re-irradiation for patients whose tumors recur less than 1 year after the initial radiotherapy. Several studies have reported a partial response rate of approximately 64-75% for induction chemotherapy with gemcitabine in combination with cisplatin prior to re-irradiation 20-23. Wang et al. also found that a regimen of induction chemotherapy with cisplatin, fluorouracil, and paclitaxel resulted in a high response rate of 66.7% for locally recurrent NPC 24. However, re-irradiation combined with chemotherapy may result in increased toxicities 10,25. Therefore, a prospective multicenter Phase 3 randomized clinical trial is needed to investigate the efficacy and safety of induction chemotherapy in combination with radiation therapy in locally advanced recurrent NPC.

NPC is thought to be a highly immuno-inflammatory tumor associated with EBV infection, dense lymphocyte infiltration, and high expression of PDL-1 13. Chemotherapy may enhance anti-tumor immunity by promoting antigen presentation, enhancing T cell response and trafficking, alleviating immunosuppression in the tumor microenvironment (TME), and inducing immunogenic cell death 26-28. Thus, PD-1 inhibitors combined with chemotherapy have a synergistic anti-tumor effect 29,30. Over the past few years, a growing body of literature has reported the role of anti-PD-1 checkpoint inhibitors in combination with chemotherapy in the treatment of R/M NPC. The results of multiple Phase 3 clinical trials (CAPTIN-1st, JUPITER-02, and RATIONALE 309) have shown that anti-PD-1 checkpoint inhibitors in combination with chemotherapy result in better PFS and are well tolerated 14-16. Whereas, these studies all included a mixture of local relapses and/or distant metastases. Accordingly, the role of anti-PD-1 checkpoint inhibitors in combination with chemotherapy in the treatment of locally advanced recurrent NPC remains worthy of further exploration. In the current study, we found higher tumor response rates but a lower overall survival rate in the RI group than in the IC group. This difference may be attributed to the higher incidence of nasopharyngeal necrosis caused by re-radiation than immunochemotherapy (Table 3), which is more likely to cause heavy nasopharyngeal hemorrhage, thereby increasing the risk of death and limit the survival benefit of re-irradiation.

Changing the fractionation method of radiotherapy may be a way to reduce late-stage complications associated with radiotherapy and improve survival in recurrent NPC patients. Recently, You et al. conducted a prospective phase Ⅲ clinical study of hyperfractionated IMRT compared with conventionally fractionated IMRT in the treatment of locally advanced recurrent NPC 31. The study showed that hyperfractionated IMRT significantly reduced the late toxicity of re-irradiation in patients with recurrent nasopharyngeal carcinoma compared with conventionally fractionated IMRT (34% vs. 57%, P = 0.023) and improved the 3-year overall survival rate (74.6% vs. 55%, P = 0.014) and quality of life of patients. This research suggests that hyperfractionated IMRT can be used as a standard treatment for locally advanced recurrent NPC. In our study, 3 of the 24 patients in the RI group received hyperfractionated IMRT. Unfortunately, two patients died of nasopharyngeal necrosis. Hyperfractionation did not show a significant survival benefit in our study, which could be related to the small sample size in this retrospective study.

## Conclusion

Taken together, re-irradiation is an effective treatment for unresectable locally advanced NPC, but the survival benefits are usually offset by severe late complications. For these patients, an anti-PD-1 checkpoint inhibitor combined with chemotherapy is a potentially effective treatment option. Future research could focus on identifying specific biomarkers that predict the immune response in order to select patients who can benefit from the immunotherapy and avoid the late complications of re-irradiation. In addition, this study was a retrospective study with a small sample size. Further randomized controlled trials in phase Ⅲ are warranted to clarify the efficacy and safety of immunotherapy in combination with chemotherapy in the treatment of recurrent, unresectable NPC.

## Figures and Tables

**Table 1:** Patient and disease characteristics

Characteristic	RI (n=24)	IC (n=18)	*P* value
Age (years)	57.3±9.6	61.3±10.2	0.199
Gender			0.508
Female	9 (37.5%)	5 (27.8%)	
Male	15 (62.5%)	13 (72.2%)	
KPS			1.0
≥80	22 (91.7%)	16 (88.9%)	
<80	2 (8.3%)	2 (11.1%)	
Histology			0.624
WHO Ⅰ	2 (8.3%)	3 (16.7%)	
WHO Ⅱ	3 (12.5%)	3 (16.7%)	
WHO Ⅲ	19 (79.2%)	12 (66.7%)	
Primary T stage			0.024
T1-2	8 (33.3%)	0 (0.0%)	
T3-4	13 (54.2%)	15 (83.3%)	
Unknown	3 (12.5%)	3 (16.7%)	
Primary N stage			0.815
N0-1	8 (33.3%)	7 (38.9%)	
N2-3	13 (54.2%)	8 (44.4%)	
Unknown	3 (12.5%)	3 (16.7%)	
Primary overall stage			0.205
Ⅱ	5 (20.8%)	0 (0.0%)	
Ⅲ	8 (33.3%)	9 (50.0%)	
IVA	8 (33.3%)	6 (33.3%)	
Unknown	3 (12.5%)	3 (16.7%)	
TI (months)			0.362
≤24	5 (20.8%)	6 (33.3%)	
>24	19 (79.2%)	12 (66.7%)	
Recurrent T stage			0.028
T3	16 (66.7%)	5 (27.8%)	
T4	8 (33.3%)	13 (72.2%)	
Recurrent N stage			0.679
N0	19 (79.2%)	16 (88.9%)	
N1-2	5 (20.8%)	2 (11.1%)	
Recurrent overall stage			0.028
Ⅲ	16 (66.7%)	5 (27.8%)	
ⅣA	8 (33.3%)	13 (72.2%)	
EB DNA copies			0.121
≤500	17 (70.8%)	17 (94.4%)	
>500	4 (16.7%)	0 (0.0)	
Unknown	3 (12.5%)	1 (5.6%)	

RI: Re-irradiation group; IC: Immunochemotherapy group

**Table 2:** Tumor response in Re-irradiation group or Immunochemotherapy group

	Group		χ^2^	*P*
Response	RI	IC		
CR	6 (25.0%)	2 (11.1%)	7.564	0.023
PR	16 (66.7%)	8 (44.4%)		
SD	2 (8.3%)	8 (44.4%)		

CR, complete response; PR, partial response; RI: Re-irradiation group; IC: Immunochemotherapy group

**Table 3:** Frequency of acute toxicities from the two groups by type and grade

Event	RI (N=24)		IC (N=18)	
	Any Grade n (%)	Grade≥3 n (%)	Any Grade n (%)	Grade≥3 n (%)
Acute toxicity				
Leucopenia	11 (45.8%)	3 (12.5%)	14 (77.8%)	4 (22.2%)
Neutropenia	9 (37.5%)	1 (4.2%)	8 (44.4%)	4 (22.2%)
Thrombocytopenia	8 (33.3%)	0	10 (55.6%)	2 (11.1%)
Anemia	15 (62.5%)	0	16 (88.9%)	1 (5.6%)
ALT elevation	5 (20.8%)	1 (4.2%)	4 (22.2%)	0
AST elevation	5 (20.8%)	0	2 (11.1%)	0
Total bilirubin elevation	3 (12.5%)	0	2 (11.1%)	0
Direct bilirubin elevation	0	0	0	0
Blood cholesterol elevation	4 (16.7%)	1 (4.2%)	1 (5.6%)	0
Blood triglyceride elevation	9 (37.5%)	1 (4.2%)	5 (27.8%)	0
Serum creatinine elevation	1 (4.2%)	0	0	0
Creatine kinase elevation	4 (16.7%)	0	1 (5.6%)	0
Hypertension	0	0	0	0
Hypothyroidism	3 (12.5%)	0	4 (22.2%)	0
Hyperthyroidism	0	0	1 (5.6%)	0
Nausea	15 (62.5%)	0	14 (77.8%)	0
Vomiting	2 (8.3%)	0	2 (11.1%)	0
Diarrhea	1 (4.2%)	0	0	0
Weight loss	7 (29.2%)	0	3 (16.7%)	0
Dermatitis	16 (66.7%)	0	1 (5.6%)	0
Stomatitis (mucostitis)	19 (79.2%)	0	0	0
Pruritus	0	0	3 (16.7%)	0
Fever	0	0	4 (22.2%)	0
Fatigue	20 (83.3%)	0	16 (88.9%)	0
Myositis or myalgia	0	0	0	0
Joint pain	0	0	1 (5.6%)	0
0Infusion reaction	0	0	0	0
Pneumonia	0	0	0	0
Late toxicity				
Xerostomia	23 (95.8%)	0	14 (77.8%)	0
Nasopharyngeal necrosis	16 (66.7%)	10 (41.7%)	4 (22.2%)	0
Deafness	4 (16.7%)	0	4 (22.2%)	0
Eye damage	2 (8.3%)	0	3 (16.7%)	0
Cranial neuropathy	3 (12.5%)	1 (4.2%)	1 (5.6%)	0
Trismus	3 (12.5%)	0	1 (5.6%)	0
Temporal lobe necrosis	3 (12.5%)	0	2 (11.1%)	0

RI: Re-irradiation group; IC: Immunochemotherapy group

**Table 4:** Effect of prognostic factors on survival in univariate analyses

Factors	3y-OS			3y-PFS			3y-LRFS			3y-DMFS (%)	
	%	*P*		%	*P*		%	*P*		%	*P*
Gender		0.869			0.443			0.194			0.558
Male	32.4			60.3			68.2			91.9	
Female	39.7			41.4			41.4			100.0	
Age (years)		0.406			0.847			0.864			0.969
≤60	0.455			53.7			59.8			93.9	
>60	0.247			52.9			56.3			95.1	
Histology		0.386			0.620			0.365			0.619
WHOⅠ	28.6			NA			NA			100.0	
WHOⅡ	22.2			100.0			100.0			100.0	
WHO Ⅲ	37.8			49.4			55.9			92.8	
Primary T stage		0.595			0.262			0.242			0.866
T1-2	12.5			45.0			60.0			83.3	
T3-4	45.8			49.9			49.9			100.0	
Unknown	33.3			83.3			100.0			83.3	
Primary N stage		0.164			0.872			0.951			0.627
N0-1	38.5			59.6			67.7			92.3	
N2-3	35.5			40.9			40.9			100.0	
Unknown	33.3			83.3			100.0			83.3	
Primary overall stage		0.569			0.379			0.217			0.394
Ⅱ	20.0			51.9			77.8			75.0	
Ⅲ	38.1			51.0			51.0			100.0	
ⅣA	43.8			44.0			44.0			100.0	
Unknown	33.3			83.3			100.0			83.3	
TI (months)		0.649			0.843			0.844			0.614
≤24	29.5			53.3			53.3			100.0	
>24	36.0			53.2			60.0			92.7	
Recurrent T stage		0.255			0.360			0.733			0.471
T3	23.0			40.9			50.1			89.1	
T4	54.0			69.0			69.0			100.0	
Recurrent N stage		0.583			0.736			0.497			0.693
N0	34.2			55.5			61.6			93.5	
N1-2	40.8			39.5			39.5			100.0	
Recurrent overall stage		0.255			0.360			0.733			0.471
Ⅲ	23.0			40.9			50.1			89.1	
ⅣA	54.0			69.0			69.0			100.0	
EBV DNA copies		0.870			0.118			0.056			0.690
≤500	34.8			62.0			68.2			93.3	
>500	45.0			0			NA			100.0	
Unknown	37.5			42.9			42.9				
Regimen		0.042			0.482			0.891			0.489
RI	23.3			45.3			54.4			89.8	
IC	59.6			62.6			62.6			100.0	

OS, overall survival; PFS, progression-free survival; LRFS, local relapse-free survival; RRFS, regional relapse-free survival; DMFS, distant metastasis-free survival; RI, re-irradiation group; IC, immunochemotherapy group

**Table 5:** Impact of prognostic factors on treatment results by multivariate analysis for OS (P value)

Factors	Hazard	*P*	95% CI
Gender			
Male vs. Female	0.917	0.863	0.342 to 2.460
Age			
≤60 vs. >60	1.978	0.165	0.756 to 5.177
Regimen			
RI vs. IC	0.329	0.044	0.112 to 0.970
rOverall stage			
Ⅲ vs.ⅣA	0.728	0.522	0.275 to 1.924
EBV DNA copies			
N0 vs. N1-2	0.939	0.865	0.451 to 1.953

## Abbreviations

NPC: nasopharyngeal carcinoma
OS: overall survival
PFS: progression-free survival
LRFS: local recurrence-free survival
DMFS: distant metastasis-free survival
IC: immunochemotherapy
IMRT: intensity-modulated radiotherapy
ENPG: endoscopic nasopharyngectomy
OAR: organ at risk
PD-1: programmed cell death ligand 1
R/M: recurrent or metastasis
TOMO: tomotherapy
MRI: magnetic resonance imaging
GTVnx: gross tumor volume of the nasopharynx
CTV: clinical tumor volume
PTV: planning target volume
DFI: disease-free interval
ORR: objective response rate
RECIST: response evaluation criteria in solid tumors
RTOG: radiation therapy oncology group
CTCAE: common terminology criteria for adverse events
EBV: Epstein-Barr virus
RI: re-irradiation
CR: complete response
PR: partial response
SD: stable disease
PD: disease progression
TME: tumor microenvironment
